# Genome dynamics of *Bartonella grahamii *in micro-populations of woodland rodents

**DOI:** 10.1186/1471-2164-11-152

**Published:** 2010-03-04

**Authors:** Eva C Berglund, Christian Ehrenborg, Olga Vinnere Pettersson, Fredrik Granberg, Kristina Näslund, Martin Holmberg, Siv GE Andersson

**Affiliations:** 1 Department of Moleculcar Evolution, Norbyvägen 18C, S-752 36 Uppsala, Sweden; 2 Department of Medical Sciences, Section for Infectious Diseases, Uppsala University Hospital, S-751 85 Uppsala, Sweden; 3 Current address: Department of Microbiology, The Swedish Agricultural University, Uppsala, Sweden; 4 Current address: Socialstyrelsen, Stockholm, Sweden

## Abstract

**Background:**

Rodents represent a high-risk reservoir for the emergence of new human pathogens. The recent completion of the 2.3 Mb genome of *Bartonella grahamii*, one of the most prevalent blood-borne bacteria in wild rodents, revealed a higher abundance of genes for host-cell interaction systems than in the genomes of closely related human pathogens. The sequence variability within the global *B. grahamii *population was recently investigated by multi locus sequence typing, but no study on the variability of putative host-cell interaction systems has been performed.

**Results:**

To study the population dynamics of *B. grahamii*, we analyzed the genomic diversity on a whole-genome scale of 27 *B. grahamii *strains isolated from four different species of wild rodents in three geographic locations separated by less than 30 km. Even using highly variable spacer regions, only 3 sequence types were identified. This low sequence diversity contrasted with a high variability in genome content. Microarray comparative genome hybridizations identified genes for outer surface proteins, including a repeated region containing the *fha *gene for filamentous hemaggluttinin and a plasmid that encodes a type IV secretion system, as the most variable. The estimated generation times in liquid culture medium for a subset of strains ranged from 5 to 22 hours, but did not correlate with sequence type or presence/absence patterns of the *fha *gene or the plasmid.

**Conclusion:**

Our study has revealed a geographic microstructure of *B. grahamii *in wild rodents. Despite near-identity in nucleotide sequence, major differences were observed in gene presence/absence patterns that did not segregate with host species. This suggests that genetically similar strains can infect a range of different hosts.

## Background

Emerging infectious diseases have increased significantly during recent decades, with major implications for human health and global economy [1]. A majority has been caused by bacteria, and many of these are zoonotic, *i.e*. they are accidentally transmitted to humans from other animal reservoirs in which they do not cause disease [1]. Bacteria of the genus *Bartonella *represent an excellent model system for studies of host adaptation patterns in zoonotic agents. *Bartonella *infect the red blood cells of a wide variety of mammals, and are transmitted among hosts by blood-sucking arthropods. The genus contains two recognized human-specific pathogens, *Bartonella bacilliformis *and *Bartonella quintana*, the causative agents of Carrion's disease and trench fever, respectively. *Bartonella henselae *is naturally adapted to felines but can incidentally infect humans, manifested as cat-scratch disease. Recently, an increasing number of *Bartonella *species with natural animal reservoirs have been associated with disease manifestations in humans [2].

Rodent-associated *Bartonella *are particularly attractive for studies of emerging infectious diseases, given the abundance of rodents in nature, their importance as a reservoir for infectious diseases [3] and the observation that they carry several pathogenic *Bartonella *species [4-9]. Field studies in many different countries have shown that *Bartonella *is present in most rodent populations, with conflicting results regarding host specificity [10-18]. These studies have revealed that *Bartonella grahamii *is one of the most prevalent *Bartonella *species in wild rodents. *B. grahamii *infects many species of mice and voles, is transmitted by the rodent flea *Ctenophthalmus nobili *[19] and has been associated with two reported cases of human disease [5,6].

The genome of *B. grahamii *strain as4aup, isolated from a wood mouse in Sweden, was recently sequenced and found to consist of a 2.3 Mb circular chromosome and a 28 kb plasmid, pBGR3 [20]. This genome is slightly smaller than the 2.6 Mb genome of the closely related rat-associated species *Bartonella tribocorum *[21], but larger than the genomes of the three major human pathogens [22] (TIGR, unpublished). Sixteen genomic islands (BgGI 1-16), containing many genes for type IV and V secretion systems and phage genes, were identified in *B. grahamii *by comparison to other published *Bartonella *genomes [20]. Many of these genes are also present in genomic islands in *B. henselae*, albeit in lower copy numbers. Comparative microarray hybridizations of a global collection of *B. henselae *strains and one strain of its close relative *Bartonella koehlerae*, showed that genomic islands have been lost independently from different lineages [23,24]. Pulsed-field gel electrophoresis of the *B. henselae *strains revealed numerous rearrangements across the terminus of replication with breakpoints in regions encompassing the genomic islands [23]. In addition to these genome-scale analyses, the intra-species diversity has been studied by multi locus sequence typing (MLST) or sequencing of variable spacer regions (MST) in *B. henselae*, *B. quintana *and *B. grahamii *[23,25-29].

MLST schemes have been developed for all major human pathogens, and it has been the standard technique for classification of bacterial isolates [30,31]. However, while being useful for inferring the population structure, MLST only rarely correlates with virulence properties [32]. In contrast, microarray-based studies offer the opportunity to analyze the complete genome of closely related strains, and have been applied to study the diversity and the difference in virulence properties of many bacterial pathogens [33]. For both types of analysis, the standard has been to include a diverse set of strains, often collected at different time points in different parts of the world. While this strategy does well in grasping as much variability as possible, it gives a very limited understanding of the mechanisms and rates whereby hyper-variable sequences evolve, and of the environmental factors influencing these processes.

In this study, we have used a collection of *B. grahamii *strains isolated from wild rodents in three adjacent but non-contiguous geographic areas to analyze the genome content and the geographic microstructure of natural bacterial populations. The results revealed a geographic pattern of these strains, with few SNPs contrasting with dramatic variability in genome structure and repertoire of secretion systems.

## Results

We have analyzed 27 *B. grahamii *isolates collected from three geographic sites, separated by circa 30 km, in central Sweden (Table 1) [17]. Fourteen isolates were sampled in Håtunaholm, nine in Kumla and four in Ålbo. Sixteen were obtained from yellow-necked mice (*Apodemus flavicollis*), five from each of wood mice (*Apodemus sylvaticus*) and bank voles (*Myodes glareolus*), and one from a house mouse (*Mus musculus*). The prevalence of *B. grahamii *in the 236 captured rodents ranged between 8-16% among sites, and 6-20% among host species [17].

**Table 1:** Host, geographic origin, allelic variants and ST of the 27 *B. grahamii *strains analyzed.

Strain	Host	Origin^a^	Allelic variant^b^				ST
				
			4	7	8	15	
mm3up	*M. musculus*	Håtunaholm	1	1	1	1	1
as4aup^c^	*A. sylvaticus*	Håtunaholm	1	1	1	1	1
as4bup	*A. sylvaticus*	Håtunaholm	1	1	1	1	1
af9up	*A. flavicollis*	Håtunaholm	1	1	1	1	1
af30up	*A. flavicollis*	Håtunaholm	1	1	1	1	1
af43up	*A. flavicollis*	Håtunaholm	1	1	1	1	1
af47up	*A. flavicollis*	Håtunaholm	1	1	1	1	1
af50up	*A. flavicollis*	Håtunaholm	1	1	1	1	1
cg60up	*M. glareolus^d^*	Håtunaholm	1	1	1	1	1
cg64up	*M. glareolus*	Håtunaholm	1	1	1	1	1
af66up	*A. flavicollis*	Håtunaholm	1	1	1	1	1
af68up	*A. flavicollis*	Håtunaholm	1	1	1	1	1
cg90up	*M. glareolus*	Håtunaholm	1	1	1	1	1
af115up	*A. flavicollis*	Kumla	1	1	1	1	1
cg120up	*M. glareolus*	Kumla	2	2	2	2	2
as134up	*A. sylvaticus*	Kumla	2	2	1	2	3
af140up	*A. flavicollis*	Kumla	2	2	2	2	2
af144up	*A. flavicollis*	Kumla	2	2	2	2	2
cg147up	*M. glareolus*	Kumla	1	1	1	1	1
af156up	*A. flavicollis*	Håtunaholm	1	1	1	1	1
af163up	*A. flavicollis*	Kumla	2	2	2	2	2
af164up	*A. flavicollis*	Kumla	2	2	2	2	2
af165up	*A. flavicollis*	Kumla	2	2	2	2	2
af206up	*A. flavicollis*	Ålbo	2	2	1	2	3
as211up	*A. sylvaticus*	Ålbo	2	2	2	2	2
as224up	*A. sylvaticus*	Ålbo	2	2	2	2	2
af233up	*A. flavicollis*	Ålbo	2	2	1	2	3

^a^A map of the sampling sites is shown in Figure 5.

^b ^Loci are numbered as shown in Table 2. Only loci with polymorphisms are shown.

^c ^*B. grahamii *as4aup is the sequenced strain. Strains as4aup and as4bup are different isolates from the same host.

^d ^*Myodes glareolus *was formerly called *Clethrionomys glareolus*.

### Few sequence polymorphisms in geographically adjacent *B. grahamii *populations

Partial sequencing of four housekeeping genes (*gltA, ftsZ, batR *and *cycK*) revealed only a single polymorphism, located in *ftsZ *(Table 1, Table 2). To further differentiate the strains, we sequenced 13 loci expected to be more variable (Table 2), most of which correspond to intergenic regions that were successfully used for typing of *B. henselae *strains [28,29]. Polymorphisms were observed in only three of these regions (Table 1). In total, there were 5 SNPs in the 12 kb alignment, with three distinct sequence types (STs). We observed no sign of host specificity, for example the ST1-strains were sampled from all four host species (Table 1). In contrast, the results indicate some degree of geographic pattern, with all strains from Håtunaholm belonging to ST1, together with strains af115up and cg147up from Kumla. ST2 and ST3 were present in both Kumla and Ålbo.

**Table 2:** Characteristics of the sequenced loci and primers used for PCR.

**No**.	Locus^a^	Position^b ^and name or sequence (5'-3') of primers	Size (bp)^c^	No. of alleles^d^
1	Bgr_05150 (*cycK*)	622901 GCGCTGCTTACTTTTTCCC	580	1
		623480 TCTTTCCCCATAGATCCGC		
2	Bgr_00610 (*batR*)	82636 CAATGGTGCGATCATCTACG	535	1
		83190 CGTCTTTATCTTTTGCGCTTG		
3	Bgr_07230 (*gltA*)	872963 BhCS.1137n [61]	1039	1
		874001 CS140f [60]		
4	Bgr_13910 (*ftsZ*)	1557200 Bh ftsZ 1754.n [66]	817	2 (1)
		1558016 Bh ftsZ 965.p [66]		
5	Bgr_00430	61980 ATGCACAGCTTTCTGGTCG	590	1
		62569 TCCTGCAATAAAACCATTTGC		
6	Bgr_02350 (*phoH*)	334448 ATCAAAACAACTTGGCTCGG	692 (158)	1
	Bgr_02360	335139 TTCAGGCGATTTCATTGTAGG		
7	Bgr_03900 (*aldA*)	473807 TGTTTTCCATTTTTGAAACGC	725 (469)	2 (1)
	Bgr_03910 (*ftsK1*)	474531 CTTCTCTTGATGCACCTTTCG		
8	Bgr_05600 (*cspA*)	679952 TGAATCCGAAACCTTTTGTTG	617 (245)	2 (1)
	Bgr_05610 (*carB*)	680568 TTGGCTTTTCTGTTGTCGC		
9	Bgr_06060 (*pssA*)	734202 TAGGCGCTCTTGGTTTGG	727 (70)	1
	Bgr_06070	734928 TGGACGAGCCATTCTGTTATC		
10	Bgr_06380 (*uvrC*)	776004 CAATCATCCGGTAAACCCC	768 (397)	1
	Bgr_06390	776771 TGAAATGCGTATCCGAAAAAG		
11	Bgr_11560 (*maeB2*)	1305872 TTTTCGTGATCGTGTTTTTCC	723 (189)	1
	Bgr_11570 (*acpP2*)	1306594 GCCTGTTTTAAGGCAACGAG		
12	Bgr_15900 (*asd*)	1810101 CTCCGCGATGCTCCC	666 (281)	1
	Bgr_15910	1810766 AAATCCTTCGCCCAAAGC		
13	Bgr_17890	2061896 CAACATTAGGGGGATTGGG	592 (245)	1
	Bgr_17900 (*dut*)	2062487 AGCCGTTGCGTAGTGAGG		
14	Bgr_18510 (*pgk*)	2129539 ACCCCATCACTGCTTCCTC	605 (514)	1
	Bgr_18520 (*gap*)	2130143 CGCGTTTTGGTTTGGTATG		
15	Bgr_19030 (*ftsK2*)	2203018 CAATAAGACGCGAACCTTGAG	880 (723)	2 (2)
	Bgr_19040	2203897 TCCCCCTGCAATGAGAAG		
16	Bgr_19460 (*dnaJ2*)	2239347 GCAAAGATTCGCTCTGGAAC	708 (201)	1
	Bgr_19470 (*cobS*)	2240054 ATAGCCAGAAACCATCACACG		
17	Bgr_19730	2273643 CAAGGATTTCGTGCCCC	811 (144)	1
	Bgr_19740	2274453 TTATGTTTCGCGGTTGTTCTC		

^a ^The locus_tag and gene name, if any, for the sequenced regions in *B. grahamii *as4aup. For intergenic regions, both the upstream and the downstream genes are shown.

^b^Genomic positions of primers refer to the *B. grahamii *as4aup genome.

^c ^Size of the total PCR product refer to the *B. grahamii *as4aup genome. Within parenthesis is the size of the spacer region, if any.

^d^Within parenthesis is the number of variable sites.

### Differences in gene repertoires for type IV and type V secretion systems

**Figure 1:**
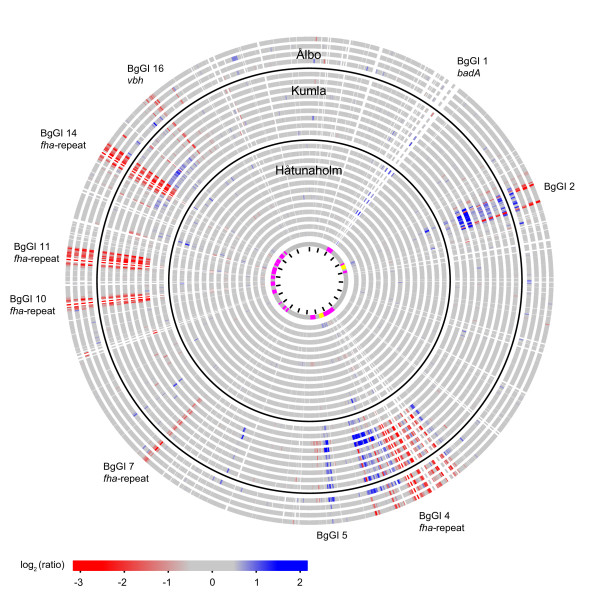
**Circular representation of the microarray results for the chromosome**. The innermost circle shows the genomic islands (BgGI 1-16) in magenta and prophages in yellow. The black lines within this circle indicate the genome position, with 100 kb between each line. Each other circle shows the CGH results for one strain, with the color corresponding to the hybridization signal relative to as4aup (red for lower and blue for higher signal according to the scale bar below the circles). Microarray probes are ordered according to the genomic position in *B. grahamii *as4aup. The reason why the *badA *region appears white is that these genes are very long, and therefore the density of probes is lower. The strains are, from inside: 1, mm3up; 2, as4bup; 3, af9up; 4, af30up; 5, af43up; 6, af47up; 7, af66up; 8, af50up; 9, af68up; 10, af156up; 11, cg60up; 12, cg64up; 13, cg90up; 14, af115up; 15, cg147up; 16, as134up; 17, cg120up; 18, af140up; 19, af144up; 20, af163up; 21, af164up; 22, af165up; 23, as224up; 24, as211up; 25, af233up; 26, af206up. Strains 1-15 are of ST1.

We screened for gene content variations using comparative genome hybridizations (CGH) to a microarray covering 96% of the genes in *B. grahamii *as4aup [20]. The results suggested that all ST1-strains have a virtually identical gene content (Figure 1), with the limitation that possible extra genes cannot be detected with CGH. All ST2 and ST3-strains displayed decreased microarray signal in comparison to the reference strain in the so-called *fha-*repeat, which is present in 5 genomic islands in the sequenced genome [20]. The *fha*/*hec *operon codes for a two-partner type V secretion system (T5SS), where the *hec *gene product probably assists in the secretion of filamentous hemagglutinin (FHA). In addition, the *fha-*repeat contains a number of putative virulence genes and phage genes [20].

The lower microarray signal in the *fha*-repeat can be due to a complete loss of these genes, a lower copy number or a high sequence divergence compared to the reference strain. Given the few identified SNPs and the observation that several other rapidly evolving genes, *e.g. badA*, did not show any indications of low signal, it is unlikely that the weak signal in the *fha*-repeat is due to sequence divergence. Attempts to amplify different *fha *genes by PCR using several sets of primers, all of which were tested successfully in the sequenced strain, resulted in one single PCR product in strain af140up and no products in strains af165up or as211up (see Additional file 1). Thus, all ST2 and ST3-strains are likely to have few or no copies of these genes.

**Figure 2:**
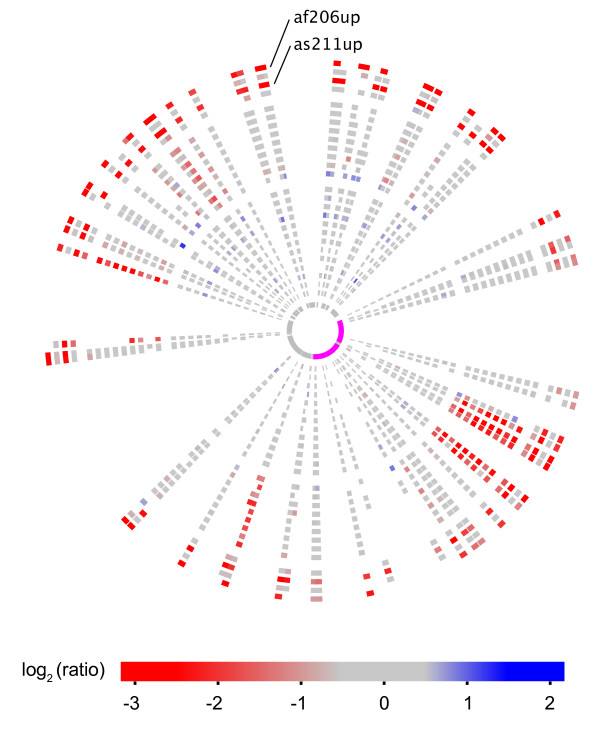
**Circular representation of the microarray results for the plasmid**. The innermost circle shows the plasmid genes (magenta indicates the *vbh *genes). The coloring and order of strains are the same as in Figure 1.

In contrast, several strains displayed a relative increase in hybridization signal in the region containing the multicopy *badA *T5SS genes (Figure 1). BadA is a trimeric autotransporter that is essential for adhesion to host cells and bloodstream infection in *Bartonella *species [21,34-37]. Since the *badA *region is already much longer in *B. grahamii *than in any other sequenced *Bartonella *genome [20], the finding that some strains seem to have additional copies of this gene was unexpected. Variability was also observed in one of the type IV secretion systems (T4SS), namely the plasmid-encoded *vbh *T4SS (Figure 2). The entire plasmid appeared to be absent from the strains af206up (ST2) and as211up (ST3) from Ålbo (Figure 2).

The ST2 and ST3-strains also displayed reduced hybridization signals in genes for two families of hypothetical proteins that are repeated in the genomes of *B. grahamii *and *B. tribocorum*, but have not been identified in any other sequenced *Bartonella *genome [20]. The first family (*e.g*. Bgr_15790) is present in a total of 17 copies in the sequenced strain, located adjacent to some of the *fha *genes. Homologs have mainly been identified in Gamma-proteobacteria, *e.g*. in a genomic island in *Photorhabdus luminescens*, in *Pseudomonas syringae *and *Listeria monocytogenes*, in one single copy in each genome. The second family (*e.g*. Bgr_18020) is present in 6 copies in the sequenced strain, located between *vbl*5 and *vbl*6 in the chromosomal *vbh *gene cluster in BgGI 16 and on pBGR3, as well as shortly upstream of the chromosomal *virB *system. No homologs to this gene have been found in other genera. The remaining variability is mostly due to phages, and includes loss/divergence of single phage genes, replication of complete prophages in BgGI 2 and BgGI 4 and putatively phage-derived bi-directional run-off replication around BgGI 15, in line with previous observations [20] (Figure 1).

### Genome structure diversity

**Figure 3:**
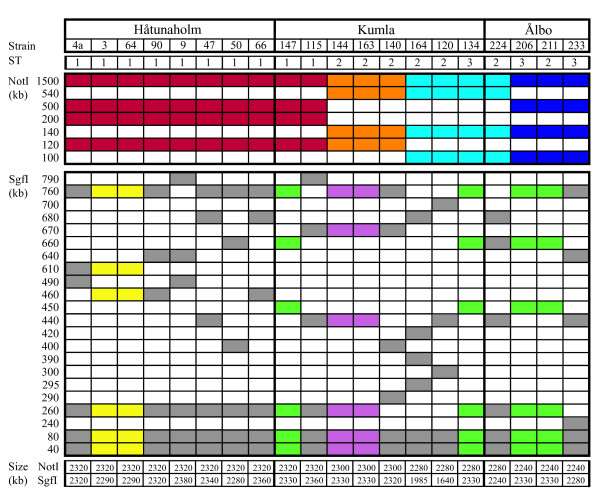
**Schematic illustration of the PFGE results**. Geographic origin, sequence type (ST) and the numbers in the strain names are shown on top. Below are the estimated sizes of all bands retrieved with the NotI and SgfI enzymes. Strains with the same pattern are shown in the same color (except grey). At the bottom are estimated genome sizes. Since the SgfI 80 kb band was shown to be a double band in the sequenced strain [20], and these parts of the genome appear to be well conserved in *B. grahamii*, this band was assumed to be a double band in all strains in the genome size estimation. Strains af164up and cg120up probably have additional double bands, or undetected bands, in the SgfI restriction since the size estimates differ a lot between SgfI and NotI.

**Figure 4:**
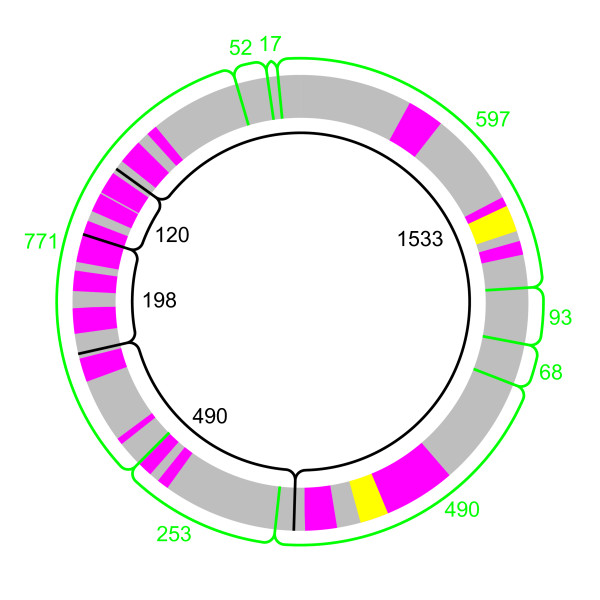
**Predicted restriction sites of NotI and SgfI in *B. grahamii *as4aup**. The circle represents the genome of *B. grahamii *strain as4aup, with genomic islands in magenta and prophages in yellow. Restriction sites are shown in black (NotI) and green (SgfI). The predicted size of each band is shown inside the circle for NotI and outside for SgfI.

Pulsed-field gel electrophoresis (PFGE) with the NotI enzyme revealed four restriction patterns in 20 representative strains and a relatively homogenous genome size of the isolates (Figure 3). One pattern was shared by all ST1-strains, two were unique to three strains from Kumla and Ålbo, respectively, and the last was shared between the remaining strains from Kumla and Ålbo. This result suggests some degree of correlation between genome structure and geographic origin. However, one of the restriction sites for the largest NotI band is located very close to the terminus of replication (Figure 4), and rearrangements around the terminus may thus have escaped detection. Therefore, we also employed the SgfI enzyme, which has more restriction sites than NotI in the sequenced genome of *B. grahamii *strain as4aup. With this enzyme, we observed a much greater diversity with 15 distinct patterns that showed no obvious correlation with geographic origin (Figure 3). This suggests that genome rearrangements occur frequently, in line with previous observations in *B. henselae *and *B. quintana *[23,27].

The 490 and 610 kb fragments obtained after cleavage of the sequenced strain with the SgfI enzyme were found to be the least conserved in the other strains (Figure 3). Both these bands contain one copy of the main prophage (Figure 4). The 490 kb band also contains several copies of the *fha*-repeat in BgGI 4 and some additional phage genes. Rearrangements within and across duplicated genes in these islands as well as across the terminus of replication could explain the variability observed after digestion with the SgfI enzyme. The other copies of the *fha*-repeat are located in BgGI 7, BgGI 10, BgGI 11 and BgGI 14. The three latter are encompassed in the 760 kb fragment of the SgfI digest and in the 120 kb and 200 kb bands of the NotI digest of strain as4aup. It is interesting to note that the 120 kb and 200 kb bands are identical in size in all ST1 strains that contain the *fha*-repeat but differ in the ST2 and ST3-strains, which have no or fewer copies of these genes.

### Genomotypes do not have a random geographical distribution

**Figure 5:**
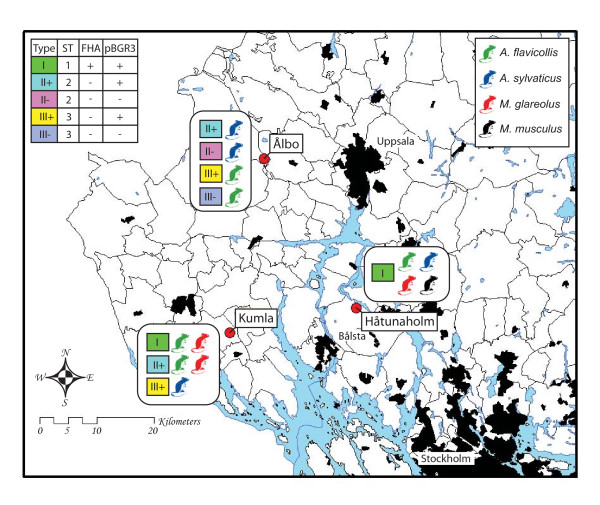
**Distribution of genomotypes across geographic sites**. The map shows the locations of the geographic sites where the rodents were collected. The definition of the genomotypes (based on sequence type, presence/absence of the *fha*-repeat and presence/absence of the plasmid) is shown in the upper left corner. For each genomotype at each site, the different rodent hosts associated with this particular variant are shown, color-coded according to the legend in the upper right corner.

We defined the genomotype based on the ST and the two major gene content variations: presence/absence of the *fha*-repeat and presence/absence of the plasmid, where absence also includes cases of lower copy number. Figure 5 summarizes the distribution of the genomotypes across geographical sites and the rodent hosts associated with each type. The most striking result is that only one genomotype was recovered from Håtunaholm (type I, which is of ST1 and harbors both the *fha*-repeat and the plasmid), although these isolates were obtained from all four different rodent host species. Genomotype I was also present in two isolates from Kumla, while absent in Ålbo. A more diverse set of isolates was identified in Kumla and Ålbo with three and four genomotypes, respectively. The *fha*-repeat displayed low signal in all ST2 and ST3-strains, suggesting that it was lost or gained only once. In contrast, the plasmid was variably present in both ST2 and ST3 and is likely to have experienced a minimum of two gain/loss events, unless one of the variants is the result of a recombination event.

### *B. grahamii *strains exhibit large differences in growth rate in liquid culture

**Figure 6:**
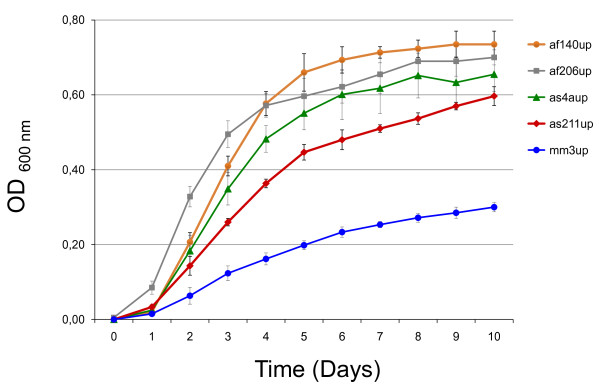
**Growth curves of *B. grahamii***. Growth curves of five *B. grahamii *strains in supplemented Schneider's medium. Bacterial growth was determined by measuring the OD_600 _in triplicates at 24-h intervals.

In order to compare genomic differences to phenotypic traits, we selected five representative strains and studied their growth in liquid culture for ten days (Figure 6). We observed large differences in growth rate, with the only strain isolated from *Mus musculus *(mm3up) growing significantly slower than the others and reaching a lower maximal optical density (Table 3). The maximal generation times, ranging from approximately 5 (as206up) to 22 (mm3up) hours, did not correlate with ST, presence/absence of the *fha*-repeat or presence/absence of the plasmid. We also performed aggregation assays for three strains (as4aup, af165up and af206up), but observed no significant differences between them (data not shown).

**Table 3:** Maximum growth rates in supplemented Schneider's medium.

Strain	ST	FHA	pBGR3	Generation time (hours)
af206up	3	-	-	5.4
af140up	2	-	+	7.0
as4aup	1	+	+	8.2
as211up	2	-	-	11.9
mm3up	1	+	+	21.8

## Discussion

In this study, we have analyzed the genomic diversity of *B. grahamii *from wild rodents in three adjacent geographic areas in central Sweden. To our knowledge, this is the first genome-scale analysis of bacterial populations from wild animals in a geographically restricted area. Since these rodents typically have a home area of less than 1 hectare, and seldom migrate distances longer than 30 km [38,39], genetic exchange between the three sites is expected to be limited. Indeed, we observed a geographic pattern in the distribution of genomotypes, showing that microstructures of bacterial populations inhabiting hosts with limited migration are visible also in the comparison of closely located sites. One of the sites, Håtunaholm, displayed a lower degree of genomic diversity than the others, with all isolates being of the same genomotype. In contrast, the presence of strains of ST2 and ST3 in both Kumla and Ålbo suggests that exchange has occurred at least twice between these regions. A possible reason for the clonal population in Håtunaholm is that this site is surrounded by water and only connected to Kumla and Ålbo by a narrow piece of land, where the small town Bålsta is situated (Figure 5). This environmental barrier is likely to restrict the genetic flow by isolation of hosts and vectors. The presence of two ST1-strains in Kumla suggests that the Håtunaholm population is a recent expansion of an immigrant from the Kumla region, or that the clonal variants in Håtunaholm occasionally escape the inland seawater barriers.

Despite sequencing regions expected to be among the most variable in the genome, we observed only five SNPs in 27 strains, corresponding to only three STs. As expected, this is much less diversity than what was observed in a previous MLST analysis of a global collection of 31 *B. grahamii *strains, where 10-16 variants were identified for each gene [40]. However, previous MLST analysis of 7 house-keeping genes in the likewise intracellular *Borrelia lucitaiae *collected from ticks on lizards in two nearby geographic areas identified 13 types in 16 isolates [41], suggesting that the low sequence diversity observed in our population is not necessarily a general feature of vector-borne bacterial populations in restricted geographic areas. Future studies of similar bacterial populations will reveal the degree of diversity that they typically display, and help elucidate the factors that govern these dynamics.

In light of the limited sequence diversity, the gene content of the studied populations was very dynamic, with the major differences being associated with the plasmid pBGR3 and the *fha*-repeat. Thus, gene gain and loss occur so frequently in nature that even strains sampled from the same geographic site with essentially no nucleotide sequence differences in a subset of their housekeeping genes can have major differences in gene content. This result shows that traditional MLST may not always be appropriate for inferring phenotypic or virulence properties, and illustrates the importance of a whole-genome approach when analyzing bacterial diversity. Furthermore, our results show that comparisons of genomic diversity in different geographic regions should be based on multiple strains from each location, since selecting only one or a few strains from each region for sequencing might easily underestimate the true genomic diversity. The real diversity could also be underestimated if there is considerable within-host heterogeneity and each host is represented by only one or a few strains. However, for *B. grahamii *we consider such within-host genomic diversity unlikely since a random sampling of a heterogeneous micro-population would recover more than a single genomotype from Håtunaholm.

The function of FHA is not known in *Bartonella*, but by homology inference to *Bordetella*, we assume that it acts as an adhesin and/or immunomodulator. In pathogenic *Bordetella *species, the *fhaB/fhaC *genes encode a two-partner type V secretion system, where FhaB is translocated to the cell surface with the aid of FhaC, which is homologous to Hec in *Bartonella *[42,43]. The mature FHA protein is required for adhesion to epithelial cells and has a role in apoptosis and immunosuppression [44-48]. The *fhaB/fhaC *genes are located within a well conserved single copy gene cluster in all pathogenic *Bordetella *species, however, several paralogs to *fhaB *have been identified [49]. Comparative genome hybridizations have shown no losses of *fhaB/fhaC*, but occasional losses of the *fhaB *paralogs [50-52]. In *Bordetella bronchiseptica*, it was suggested that one of the FhaB paralogs (FhaS) might be involved in host-specificity [53]. Although FhaS is not required for infection, a ∆*fhaS *strain was out-competed by the wild type during coinfection [53].

One could think of a similar situation in *Bartonella*, namely that some of the various *fha *gene copies confer host specificity and/or are advantageous only under certain conditions. Such a scenario would be consistent with their location within genomic islands that have been integrated/excised multiple times independently in *B. grahamii*, *B. henselae *and *B. tribocorum *[20,22], and with the many cases of pseudogenization of individual *fha *genes. Some degree of variability was observed among strains in *B. henselae *although none had completely lost the *fha *genes [23]. In contrast, the *fha *genes have been lost from all strains of *B. quintana *investigated (data not shown) and are also absent from *B. bacilliformis *[20,22]. The absence of the *fha *gene in several strains and species suggests that the gene may be lost from individual *Bartonella *populations, both temporarily and permanently, and is therefore not essential for growth and infection *per se*. Indeed, we observed no significant differences in either growth rate or level of autoaggregation between strains with different copy numbers of the *fha *gene.

Since rodents have an adaptive immune system, shedding of surface-exposed proteins could potentially enable super-infection, *i.e*. a second infection of a previously infected animal. Immunity against super-infection of the same *Bartonella *species has indeed been demonstrated in rodent hosts [54]. Under this scenario, loss of the *fha *gene might be the only way to escape acquired immunity in the rodent host population. If selection for functionality is balanced by counter-selection from the host immune system, this could trigger a periodic cycle in which strains with and without *fha *repeatedly out-compete each other. The possibility for super-infection is likely to depend on the transmission rates, and thus the abundance of hosts and vectors. A high density of hosts and vectors will increase transmission rates, and might favor loss of *fha *genes from many individuals in a population. *Vice versa*, if hosts and vectors are low in abundance, super-infections will be rare and host-acquired immunity less of a problem, which might set the stage for the return of strains with *fha*.

The only putative host-interaction genes located on the plasmid are those encoding a *vbh *T4SS that is closely related to the chromosomal *vbh *system, and more distantly related to the *virB *system, which has been shown to be essential for infection in *B. tribocorum *and secrets *Bartonella *effector proteins into the host cell [55-57]. The plasmid also contains a set of *tra *genes, including the DNA binding *traA*, which are usually involved in conjugation. It is not known whether the *vbh *gene cluster encodes proteins involved in host interactions or if it is only part of a conjugation system. The presence of a putative addiction module, consisting of a killer and an antidote protein, on pBGR3 (Bgr_p00250-Bgr_p00260) may ensure the survival of the plasmid in the population even in the absence of selection.

The observation that the Håtunaholm strains were sampled from four different rodent species shows that *B. grahamii *isolates with similar gene contents are capable of infecting diverse hosts. Such a lack of host-specificity contrasts with experimental infections of the cotton rat (*Sigmodon hispidus*) and the white-footed mouse (*Peromyscus leucopus*), which indicates that *Bartonella *strains are only able to infect hosts that are closely related to the host from which it was originally isolated [58]. Although *S. hispidus *and *P. leucopus *belong to the same family of rodents (*Cricetidae*), cross-infections were not observed [58]. The rodents sampled in Håtunaholm belong to two different families, *Cricetidae *and *Muridae*, which are genetically more diverged [59], yet harbor similar *B. grahamii *strains. This suggests a lack of host-specificity, although minor sequence differences among strains may have gone undetected in our survey. Since the only parameter showing some correlation with the generation times was the host of isolation, it is possible that the growth rate of each strain has been optimized for its particular host. It remains to be shown, however, whether a specific isolate from Håtunaholm is actually capable of infecting both mice and voles, or if there are nucleotide sequence or expression differences in outer surface proteins that account for host specificity but were not detected in this analysis.

In the future, results on the natural genomic diversity of zoonotic bacterial populations should be analyzed in an ecological context, including data about *e.g*. host and vector abundance and climate, to allow correlations between genomic diversity and bacterial prevalence or seasonal variation. Our results also urge for functional characterization of the role of FHA in *Bartonella*, and offer an opportunity to study the infectious process of strains with observed differences in genes for surface-exposed proteins in rodent hosts.

## Conclusion

Based on studies of SNPs and gene contents by comparative microarray genome hybridization, we present an illustration of how different genomotypes of *B. grahamii *are distributed across nearby geographic locations. Despite a very limited number of SNPs, we observed dramatic changes in terms of genome structure and content, with the major gains/losses being a plasmid encoding a *vbh *T4SS and a repeated region containing the *fha*/*hec *genes for a T5SS. Whereas a mixture of different genomotypes was recovered from rodents in Ålbo and Kumla, all strains isolated from rodents in Håtunaholm were identical in both ST and gene content. This suggests that environmental barriers in the form of water can lead to isolation and loss of genomic variability in host-associated bacterial populations. Neither the STs nor the gene content variability correlated with host of isolation, indicating a lack of host-specificity in these populations. However, the generation time was significantly longer for a strain isolated from *M. musculus *(more than 20 hours), than for strains isolated from *Apodemus *mice, only 5 to 12 hours. This might indicate growth rate optimization in response to the host cell environment. Given the variability in gene content and generation times for strains isolated from within such a small geographic region, we emphasize the importance of selecting more than a single strain for genomic and functional genomics analyses.

## Methods

### Bacterial strains and DNA isolation

All *B. grahamii *strains used in this study were isolated from wild rodents in September 1999 [17]. For production of bacterial stock suspensions, bacteria were harvested from hematin agar plates after four days and stored at -80°C in a buffer containing 150 mM NaCl, 50 mM Tris (pH 7) and 11.2% glycerol. The number of viable bacteria in the frozen stocks was determined by plating serial dilutions of the suspension and calculating the number of colony forming units (CFU). DNA was extracted from bacteria grown for 5-10 days on hematin agar plates, as described previously [24].

### PCR amplification

Sequence data was collected for seventeen genomic regions, using primers listed in Table 2. Primers used for PCR of *fha *are listed in Additional file 1. For *gltA *and *ftsZ*, PCR amplifications were performed with a Perkin-Elmer GeneAmp 9600 thermocycler using a PCR Master kit from Boehringer Mannheim Scandinavia AB (Bromma, Sweden). The 50 μl reaction mixture consisted of the template, forward and reverse primers (10 pmol/primer), 25 μl PCR master mix, and distilled water of PCR-grade up to 50 μl. The reaction conditions were 5 min at 95°C, 10 cycles at 94°C for 20 s, 50°C for 1 min, 72°C for 90 s, annealing temperature lowered 1°C per cycle until reaching 40°C. This was followed by 40 cycles at 94°C for 20 s, 40°C for 1 min, 72°C for 90 s and a 7 min extension period at 72°C. PCR products were purified with the QIAquick purification kit from QIAGEN, Inc., Chatsworth, Calif., U.S., according to the manufacturer's instructions. PCR products were sequenced with an ABI 310 Genetic Analyzer (Perkin-Elmer Corp., Norwalk, Conn., US). The *gltA *product was sequenced with primers CS443f [60] and BhCS.781p [61] in addition to the primers used for PCR. Sequencing reactions were performed using a DNA Sequencing Kit with AmpliTaq^® ^DNA Polymerase, FS, for the BigDye™ Terminator Cycle Sequencing Ready Reaction protocol (Perkin-Elmer, Applied Biosystems, Warrington, GB). Remaining PCR reactions were performed as described previously [23].

### Analysis of sequence data

Sequences for *gltA *and *ftsZ *were analyzed with ABI Prism™ DNA Sequencing Analysis Software Version 3.0 (PE Applied Biosystems, Foster City, CA, US) and Sequencher™ (Gene Codes Corporation, Ann Arbor, MI, US). Remaining sequences were assembled and edited with Phred, Phrap and Consed [62-64]. The allele of the sequenced strain was named allele 1 and the combination of alleles was used to define the sequence types.

### Microarray comparative genome hybridizations

Microarrays were designed and manufactured as reported previously [20] and cross-linked at 250 mJ/cm^2 ^(slides printed at the Royal Institute of Technology) or 800 mJ (slides printed at Uppsala University). Genomic DNA from the sequenced strain was used as reference in all hybridizations and two or three hybridizations were performed for each strain. Prehybridization, DNA labeling, hybridization, scanning and image analysis were performed as described previously [20]. The channel used for the reference strain is referred to as Ch1 and the test strain is referred to as Ch2, and spots meeting the following criteria were removed from further analysis: spots flagged as bad or not found during quantification, spots with more than 10% saturated pixels in either channel, Ch1 spot median below 2 times the Ch1 background median, less than 90% of pixels having a Ch1 intensity higher than background intensity plus one standard deviation, less than 75% of pixels having a Ch1 intensity higher than background intensity plus two standard deviations or spots having less than 70 pixels. M-values were computed as log_2_(Ch2/Ch1) and normalization was performed as described previously [23]. Median M-values of all replicate spots and arrays were computed for each strain. A gene was defined as absent if the median M-value of all probes in that gene was ≤ -2.

### Pulsed-field gel electrophoresis

Bacteria grown for 10 days were digested with 10 U of NotI (New England Biolabs) or SgfI (Promega) restriction endonucleases. Experimental procedures for digestion were as reported previously [24]. The DNA fragments were separated in 0.9% PFGE-grade agarose (SeaKem^® ^Gold; Cambrex Bio Science) in 0.5× TBE buffer in GenNavigator^® ^System apparatus (Amersham Biosciences) at 14°C and 5.6 V/cm, for a total of 65 hours. The total run was separated in six phases; switch times ramped from 5 to 150 s. The sizes of the fragments were estimated using PFGE λ-ladder and Yeast Chromosome PFG marker (New England Biolabs).

### Growth curves in liquid culture

For growth in liquid culture, frozen bacterial stocks were thawed on ice and inoculated at a concentration of approximately 10^6 ^CFU per ml into Schneider's insect medium (Sigma) supplemented with 10% FBS (heat inactivated at 56°C for 30 min) and 5% sucrose, as described by Riess *et al*. [65]. In addition, the medium was supplemented with 0.1 M HEPES, to keep the pH near 7. Bacterial growth was determined by measuring the optical densities at 600 nm (OD_600_) in triplicates at 24-h intervals. For strain as4aup, the number of CFU was determined at each time point by plating 10-fold serial dilutions. We used the formula described by Reiss *et al*. [65] to estimate the maximum growth rate, expressed as generation time, for each strain, assuming a similar amount of bacteria at a certain optical density as for as4aup.

### Aggregation assays

Bacteria grown in liquid cultures as described above were sampled in the mid-exponential phase. The bacterial cells were centrifuged, and pellets were suspended in phosphate buffered saline (PBS) to an OD_600 _of 0.6. After gentle vortexing, standing tubes with 5 ml of bacterial suspension were incubated at 35°C, and the OD_600 _of the upper part of the suspensions was measured throughout time (0 min, 5 min, 15 min, 1 h, 4 h and 22 h).

### Nucleotide sequence and microarray accession numbers

The novel sequences of *ftsZ *and three spacers have been submitted to GenBank under the accession numbers FN597604. FN597607. The microarray data have been deposited in the ArrayExpress database of the European Bioinformatics Institute under the accession number the accession numbers A-MEXP-1741 and A-MEXP-1576 for the array designs and E-TABM-850 for the experimental data.

## List of abbreviations

MLST: multi locus sequence typing; MST: multi spacer typing; CGH: comparative genome hybridization; PFGE: pulsed-field gel electrophoresis; SNP: single nucleotide polymorphism; ST: sequence type.

## Authors' contributions

ECB performed the microarray CGH experiments, carried out the sequence and microarray data analyses and prepared the manuscript. CE participated in the sequence analysis and drafted part of the manuscript. OVP performed the PFGE experiments. FG performed the cultivation and aggregation experiments. KN performed the cultivation and sequencing experiments. MH contributed strains, analyzed experimental data and helped in the design of the study. SGEA designed the study, analyzed experimental data, prepared the manuscript and coordinated the project. All authors read and approved the final manuscript.

## Supplementary Material

Additional file 1 List of primers used for PCR of *fha*, and PCR results. Click here for file
