# Supplementary material for: Genome dynamics of Bartonella grahamii in micro-populations of woodland rodents
Source: BMC Genomics. 2010 Mar 4;11:152. doi: 10.1186/1471-2164-11-152 (PMC2847970; doi:10.1186/1471-2164-11-152)
Supplement: Additional file 1 — List of primers used for PCR of fha, and PCR results. [file 1471-2164-11-152-S1.PDF]

Additional file 1: Primers used for PCR of *fha*.

Primer 1	Primer 2	Product		
		af140up	af165up	as211up
TTGCCAATGCAGGCG	AGCATCGACACTCTCACCG	no	no	no
AACAACCAATCATTCACGAGC	GCCAAGCTCTTTCCTCACC	no	no	no
GGCTCTACAGGCGGACAG	AGGAAGCCCCTCAGAAAGTT	yes	no	no
GCAGTGCGCTTCATGGTC	GGGTGCTCTTTGACATTTGTG	no	no	no
GTCCCAACCTTTGGGGAA	CATTGCCTTTAACCCCAACA	no	no	no
CATGGATGTTTATGCGCC	AGTTGATATTCCCGCTCTGC	no	no	no
